# Application of OpenAI GPT-4 for the retrospective detection of catheter-associated urinary tract infections in a fictitious and curated patient data set

**DOI:** 10.1017/ice.2023.189

**Published:** 2024-01

**Authors:** Jasmin Perret, Adrian Schmid

**Affiliations:** Infectious Diseases and Hospital Epidemiology, Department of General Internal Medicine, Cantonal Hospital Winterthur, Winterthur, Switzerland

## Abstract

The use of the OpenAI GPT-4 model in detecting catheter-associated urinary tract infection (CAUTI) cases in small fictitious and curated patient data sets was investigated. Final analysis of 50 patients including 11 CAUTI cases yielded sensitivity, specificity and positive and negative predictive values of 91%, 92%, 83%, and 96%, respectively.

Surveillance of hospital-acquired infections (HAIs) is one of the crucial elements of hospital infection prevention and control. Manual surveillance is tedious and extremely time-consuming. Automated surveillance could potentially offer more speed, accuracy, and efficiency. Many structural and technological challenges to fully automated infection surveillance remain and some HAIs are inherently more suited to an automated process. Further barriers to automating systems are the lack of intra- and interfacility reliability and reproducibility, missing data, need for substantial informatics support, and initial validation requirements. Some of these challenges could potentially be overcome using artificial intelligence (AI).^1^

AI Generative Pre-trained Transformer 4 (GPT-4) was made publicly available on March 14, 2023.^2^ GPT-4 has been shown to have medical capabilities, exceeding the passing score on US medical licensing examination by 20 points and outperforming earlier general-purpose models (GPT-3.5) as well as models specifically fine-tuned for medical knowledge (Med-PaLM).^3^ We applied Open accessible AI GPT 4 on a data set of a fictitious and curated patient data set to test its utility and accuracy in detecting catheter-associated urinary tract infections (CAUTIs).

## Methods

Two data sets, data set 1 and data set 2, were created using Microsoft Excel version 16.72 software (Microsoft, Redmond, WA). Both data sets contained information on fictitious patient demographics, body temperature, urine culture results (ie, name of the pathogen(s) and colony forming units), and calendar dates of admission and catheterization (ie, insertion and removal).

Data set 1 was created to represent a normal patient population in an acute-care hospital, comprising data that are usually available as structured data elements in the electronic health record (EHR), often used in semiautomated surveillance. Patients without urinary catheters were included to check whether GPT reliably excludes such patients from having a CAUTI.

Data set 2 also included information on clinical symptoms such as information on dysuria, suprapubic and/or costovertebral tenderness, and urinary urgency and/or frequency. Data set 2 was created using data set 1 and adding 6 new patients. Because the prompt length of GPT-4 is currently limited to 8,192 tokens, data set 2 had to be shortened accordingly by removing 35 patients. Data set 2 was created to check whether GPT-4 was able to include all CAUTI criteria in its analysis.

GPT-4 was first provided data set 1 and subsequently data set 2 as comma-separated values (CSV) files. GPT-4 was asked to analyze the data and to identify patients with CAUTIs based on National Healthcare Safety Network (NHSN) criteria,^4^ as well as to retrieve patient and catheter days and to calculate the catheter utilization ratio. Sensitivity, specificity, and predictive values of CAUTI detection by GPT-4 were calculated initially and after repeated training. As a gold standard, manual detection based on NHSN criteria was performed separately by an infection prevention specialist and an infectious disease doctor.

## Results

**Figure 1.**
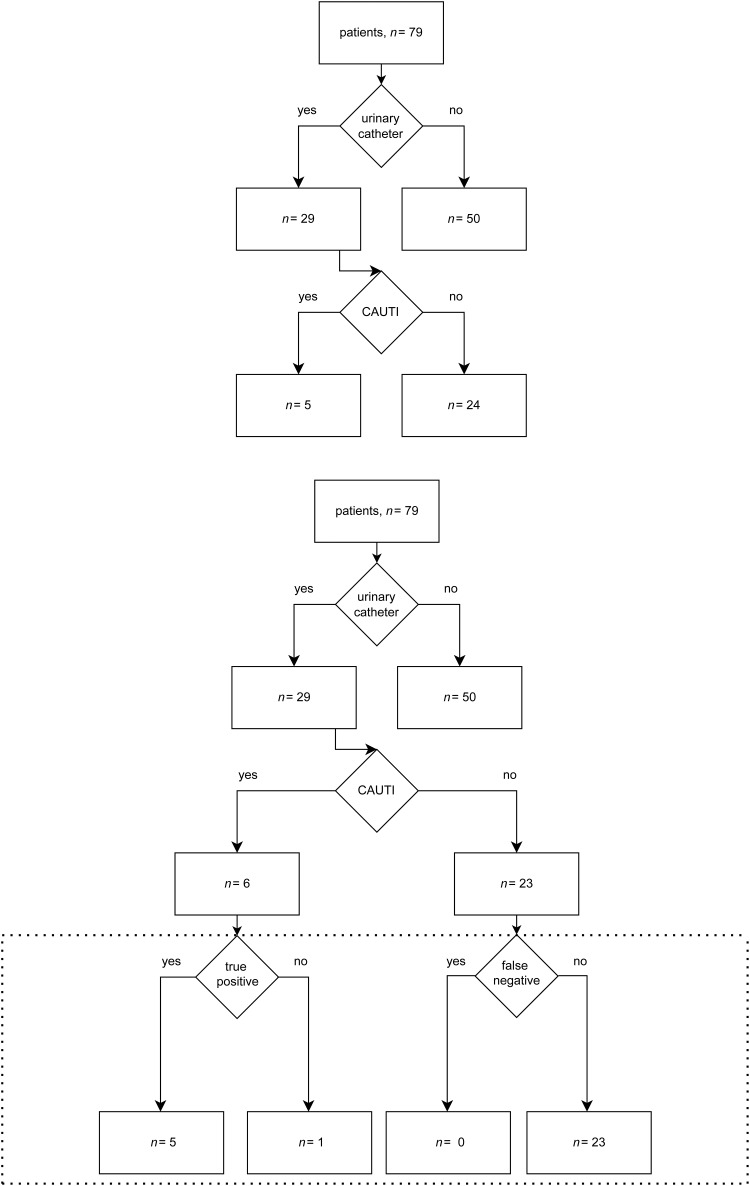
Data set 1. (Above) Manual analysis. (Below) Analysis by GPT-4. The dashed box shows a comparison of GPT-4 to manual analysis.

Data set 1 included a total of 79 patients, 29 patients with and 50 patients without indwelling urinary catheters (Fig. 1). Manual detection performed separately by an infection prevention specialist and an infectious disease doctor concordantly revealed 5 patients with a CAUTI. Data set 1 comprised 869 patient days and 114 catheter days, with a catheter utilization ratio of 13.12%.

GPT-4 initially identified 6 patients in data set 1 with CAUTIs, 4 of which met the criteria. Also, 1 patient with a CAUTI was not detected; he had the catheter removed 1 day before infection occurred. Furthermore, 2 patients were falsely identified as having CAUTIs; 1 patient had 3 different bacterial species in the urine culture, whereas the other patient did not meet all criteria within the infection window period.

The initial analysis by GPT-4 yielded sensitivity, specificity, and positive and negative predictive values of 80%, 92%, 67%, and 96%, respectively. After repeated training with ∼18 rounds of 2–25 queries each, GPT-4 correctly recognized all 5 patients with CAUTIs but still falsely reported a single case in which not all criteria were met within the infection window period. The final results using data set 1 showed sensitivity, specificity, and positive and negative predictive values of 100%, 96%, 83%, and 100%, respectively.

**Figure 2.**
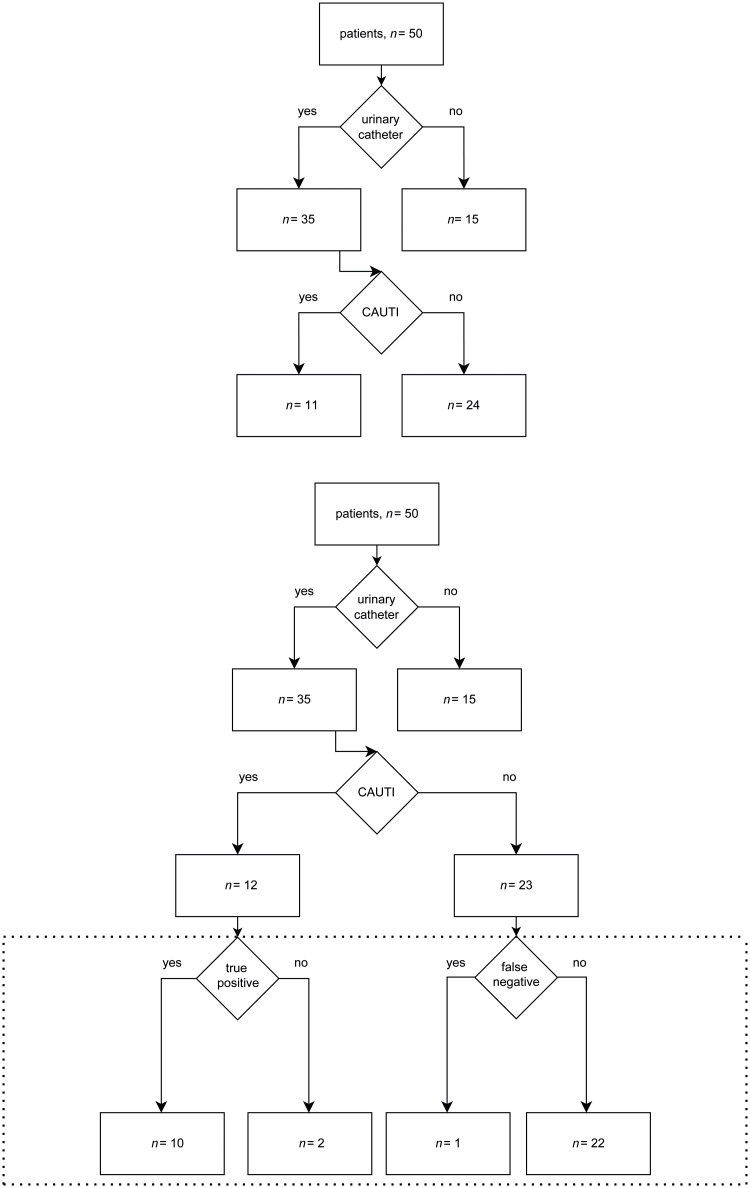
Data set 2. (Above) Manual analysis. (Below) Analysis by GPT-4. The dashed box shows a comparison of GPT-4 to manual analysis.

Data set 2 included 50 patients: 35 with and 15 without indwelling urinary catheters (Fig. 2). According to separate, concordant manual detection, 11 patients met the NHSN criteria for a CAUTI. Patient days, catheter days, and the catheter utilization ratio were 550 days, 218 days, and 39.64%, respectively.

In data set 2, GPT-4 identified 12 patients with CAUTIs, 10 of which met the criteria. One patient with a CAUTI was not detected, the same that GPT failed to detect in the first round of analysis of data set 1. Also, 2 patients were falsely identified as having a CAUTI. In one case not all criteria were met within the infection window period (identical patient as the false positive in the final analysis of data set 1), and in 1 case the urinary catheter was only in place for 1 calendar day. The latter was correctly classified in the analysis of data set 1. The analysis of data set 2 yielded sensitivity, specificity, and positive and negative predictive values of 91%, 92%, 83%, and 96%, respectively.

Patient days, catheter days, and catheter utilization ratio were correctly retrieved by GPT-4 in both data sets, but several attempts were necessary because GPT-4 encountered difficulties with data structure in this case and initially erroneously counted empty Excel cells (ie, no catheters were placed) as 1 catheter day.

## Discussion

AI-based tools, such as machine learning and natural language processing, to control HAIs have already been evaluated.^5^ One study focused on prediction of urinary tract infections with machine learning,^6^ whereas another applied natural language processing for real-time CAUTI surveillance.^7^ The latter showed an overall sensitivity of 65% and positive predictive value of 54%. Our results indicate that an OpenAI GPT-4 model could potentially be used in the surveillance of HAIs such as CAUTI.

This study had several limitations. The small number of idealized and curated patient data (limited by a prompt length of 8,192 tokens), the very high CAUTI prevalence in the second data set as well as the retrospective nature of the analysis likely constitute a bias toward positive effects. Furthermore, reliability and reproducibility of results on repeated trials was limited, difficulties arose when confronted with empty cells and the need for manual review was not replaced. Due to limited IT support, we were unable to compare GPT-4 to a specific automatic CAUTI surveillance algorithm.

Conceivably, future AI models could be employed at the interface level to analyze data directly from the EHR, enabling an efficient approach to HAI surveillance. According to recent media reports, Epic (Epic, Verona, WI) intends to integrate GPT-4 in its EHR system.^8^ The scalability of such an approach is clearly limited because not all medical centers have access to EHRs. Further barriers such as potential costs for ongoing maintenance, validation requirements, reliability, reproducibility, and missing data need to be considered.^1^ Moreover, legal issues such as data privacy and protection need to be addressed before implementation in clinical medicine.^9^

Although this technology demonstrates promise, additional work on real-world data are needed to validate results, and implementation barriers remain. The initial inability to correctly analyze empty cells highlights the importance of considering these barriers before the system can be relied upon to support HAI surveillance activities.
